# Preliminary Evolutionary Trends of *Ctenophorus* Dragon (Lacertilia: Agamidae) Lifespans, With a New Longevity Record

**DOI:** 10.1002/ece3.73191

**Published:** 2026-03-19

**Authors:** Daniel Hoops, Martin J. Whiting

**Affiliations:** ^1^ School of Natural Sciences Macquarie University Sydney New South Wales Australia

**Keywords:** annual, iteroparous, K‐selection, life history, lizard, longevity, r‐selection, semelparous

## Abstract

We reviewed the published literature on the lifespans of dragon lizards in the genus *Ctenophorus* (Agamidae) and collated all known lifespan records for this genus, both in the wild and in captivity. We found that *Ctenophorus* species for which longevity data in the wild are available are about evenly split between those that live 1 year or less (annuals) and those that live multiple years. In captivity, *Ctenophorus* are generally longer lived than in the wild. However, one annual species, the painted dragon ( *Ctenophorus pictus* ), appears to live only 1 year even in captivity, suggesting that longevity can be more constrained. We also document a new longevity record for the genus: a female rusty dragon ( *Ctenophorus rufescens* ) lived for 12 years in our care after being captured as an adult. We estimate her lifespan to have been at least 13 years. *Ctenophorus* show both a wide range of longevity and variance in the degree to which it is plastic, both of which warrant further investigation.

## Graphical abstract

A female rusty dragon ( *Ctenophorus rufescens* ) in the Musgrave Range, Northern Territory, Australia in 2012. This dragon was collected as part of a study by the authors. A different female rusty dragon collected at the same time survived 12 years in captivity, making it the oldest known *Ctenophorus* dragon. Photo by Angus Kennedy.

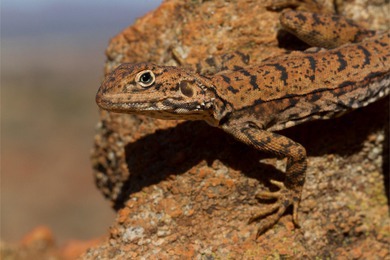

Among reptiles, agamids in the genus *Ctenophorus* are notable for their relatively short lifespans (Greer 1989a; Melville and Wilson 2019). While most reptiles live multiple years, several intensively studied *Ctenophorus* species appear to be annuals, reaching maturity, breeding, and dying in under a year. However, reports also indicate that some *Ctenophorus* species have high survivorship rates over multiple years, both in the wild and in captivity.

To develop a holistic picture of longevity in *Ctenophorus*, we conducted a compresentive systematic literature review (see our Open Science Framework repository, DOI: 10.17605/OSF.IO/8NU2X), which produced data for 17 taxa in the wild (Table 1). Of those species, ten are reported to be annuals or probably annuals. However, none of the species are very long lived. Most species that live more than a year survive only 2–3 years and only two species regularly survive 4 years or more.

**TABLE 1:** Published longevity records in wild and captive *Ctenophorus* dragons.

*Ctenophorus* species		Longevity		Source(s)
English name	Scientific name	Wild	Captive	
Western Heath Dragon	*C. adelaidensis*	2 years		1(as *Tympanocryptis adelaidensis adelaidensis*)
Western Ring‐tailed Dragon	*C. caudicinctus*	Annual^a^		2(as *Amphibolurus caudicinctus*)
Southern Heath Dragon	*C. chapmani*	2–3 years		3(as *Amphibolurus adelaidensis*)
Long‐legged Sand Dragon	*C. femoralis*	Probable Annual		4
Peninsula Dragon	*C. fionni*	1–2 years (max. 6)	10 years	5, 6
Goldfields Ring‐tailed Dragon	*C. infans*		6 years	7(as *Ctenophorus caudicinctus infans*)
Central Military Dragon	*C. isolepis gularis*	Annual		8, 9(as *Amphibolurus isolepis gularis*)
Central Military Dragon	*C. i. isolepis*	Annual		9(as *Amphibolurus isolepis isolepis*)
Spotted Sand Dragon	*C. maculatus*	Annual		9(as *Amphibolurus maculatus maculatus*)
Lake Eyre Dragon	*C. maculosus*	3–3.5 years		10(as *Amphibolurus maculosus*)
Barrier Range Dragon	*C. mirrityana*		11.5 years	11(as *Ctenophorus decresii*), 12
Swift Rock Dragon	*C. modestus*	5 years		13(as *Ctenophorus decresii*)
Central Netted Dragon	*C. nuchalis*	Annual	11 years	6, 8, 11, 14, 15(latter two as *Amphibolurus nuchalis*), 16
Ornate Dragon	*C. ornatus*	1.5 years (max. 11)		17(as *Amphibolurus ornatus*)
Painted Dragon	*C. pictus*	Annual	Annual	16, 18, 19, 20(as *Amphibolurus pictus*), 21
Rufus Sand Dragon	*C. rubens*	Probable Annual		22
Rusty Dragon	*C. rufescens*		≥ 13 years	this report
Claypan Dragon	*C. salinarum*	≥ 2 years		23
Eastern Mallee Dragon	*C. spinodomus*	Annual		24(as *Amphibolurus fordi*), 25(as *Ctenophorus fordi*), 26
Southern Mallee Dragon	*C. tuniluki*	Annual		27(as *Amphibolurus fordi*)

*Note:* 1 Bamford (1992), 2 Storr (1967), 3 Tyler (1959), 4 Greer (1989b), 5 Johnston (2011), 6 Brown (2012), 7 Bush (2024), 8 Dickman et al. (1999), 9 Storr (1965), 10 Mitchell (1973), 11 McFadden and Harlow (2007), 12 McFadden and Purcell (2015), 13 McLean et al. (2015), 14 Bradshaw (1981), 15 Bradshaw (1986), 16 Read and Owens (1999), 17 Bradshaw (1971), 18 Henle (1989), 19 Healey (2008), 20 Mayhew (1963), 21 Olsson et al. (2007), 22 Melville and Wilson (2019), 23 Chapman and Dell (1985), 24 Cogger (1969), 25 Uller and Olsson (2006), 26 Sadlier et al. (2019), 27 Baverstock (1979).

^a^ Bush (2024) disagrees that *C. caudicinctus* is an annual and hypotheses a lifespan of two years or more.

Mapping longevity onto the *Ctenophorus* phylogeny highlights the paucity of available data, with most species lacking known lifespans (Figure 1). Based on the limited data available, it appears that living multiple years is likely the ancestral state of the genus. Annual survivorship only appears in one of the two first‐order *Ctenophorus* subclades. Furthermore, annual survivorship is not phylogenetically conserved within the clade but appears to have evolved multiple times from longer lived ancestors.

**FIGURE 1:**
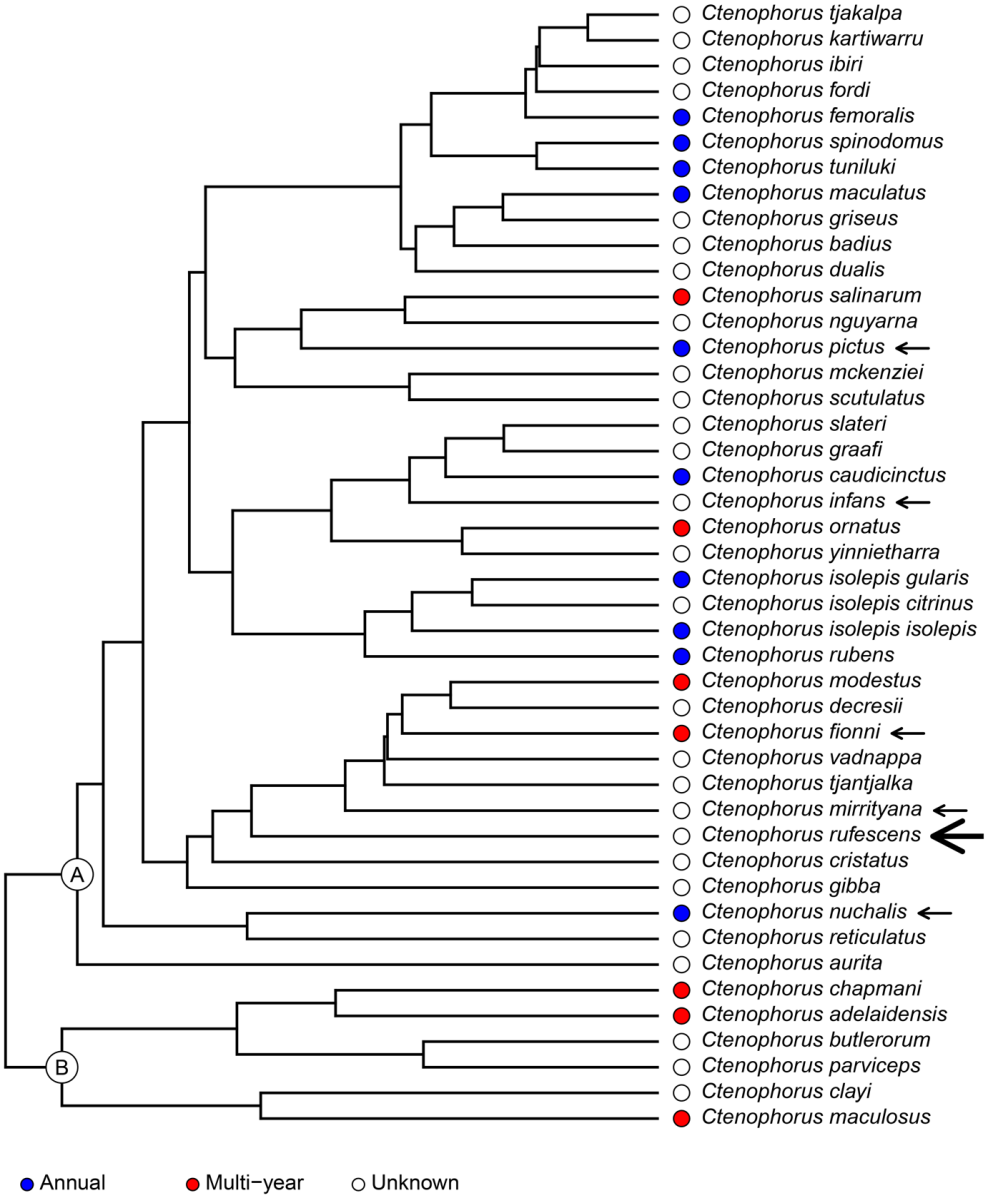
A phylogeny of all *Ctenophorus* agamid species, pruned from Brennan et al.'s (2025) phylogeny of Amphibolurine dragon lizards. The coloured dots beside each tip indicate whether the species has been reported to be annual (blue dots) or to live longer than a year (red dots), or whether we could not find any published data for the species in the wild (white dots). Arrows indicate the species for which we were able to find longevity data in captivity. The large arrow highlights the rusty dragon ( *Ctenophorus rufescens* ), the species for which we are publishing a new captive longevity record here. The two first‐order nodes within *Ctenophorus* are labelled A and B, with only clade A containing lineages that have evolved annual lifespans, suggesting that living multiple years is the *Ctenophorus* ancestral state. Furthermore, within clade A annual survivorship is not phylogenetically conserved, suggesting it evolved from longer lived ancestors multiple times within the genus. The data and code necessary to replicate this figure are available at the Open Science Framework repository for this manuscript, DOI: 10.17605/OSF.IO/8NU2X.

Our search also revealed a few reports of longevity data for *Ctenophorus* species in captivity (Table 1). These show that captive *Ctenophorus* can be, but are not always, surprisingly long‐lived. We expect that lifespans in captivity are longer than in the wild (Brown 2012), but this is not entirely supported by the species for which we were able to find both wild and captive longevity data. The central netted dragon ( *Ctenophorus nuchalis* ), an annual species in the wild, has been reported to live 10 years in captivity, indicating it can live far longer than a year given the right conditions (Table 1). In contrast, the annual painted dragon ( *Ctenophorus pictus* ) appears to only live for 1 year even in captivity, suggesting a fixed lifespan (Table 1). This limited evidence, from just two species, suggests that *Ctenophorus* species may be either facultatively or obligately annual. Captive longevity data from additional species for which we have wild longevity data (Table 1) would be ideal for testing this hypothesis. As many of these species are represented in zoos, captive research colonies, and Australian herpetoculture, it is likely that this information exists and just needs to be collated and published (e.g., see generalized comments in Brown 2012, 184 & 261).

*Ctenophorus* present an opportunity to examine the environmental and ecological factors that can drive evolutionary shifts in longevity. For example, in the wild the longest lived *Ctenophorus*, according to currently available data, are specialist rock dwelling species, i.e., saxicoline: peninsula, swift rock, and ornate dragons (Table 1). In captivity, a saxicolous species, the Barrier Range dragon ( *Ctenophorus mirrityana* ) held the longevity record prior to this publication (McFadden and Purcell 2015). This limited evidence suggests that saxicolous living may favour longer lifespans.

We are not aware of any data on the lifespan of one saxicolous species, the rusty dragon ( *Ctenophorus rufescens* ). In October 2012 DH collected rusty dragons from the northern Musgrave Ranges in the far south of the Northern Territory and brought them to the Australian National University in Canberra for study. Two females survived beyond the length of the study and were maintained in captivity. The longer‐lived individual survived until October 4, 2024, living for 12 years (Figure 2). As she was an adult when captured, we estimate that she lived to at least 13 years. After she died she was deposited in the collection of the Australian Museum in Sydney with accession number AMS R.203166.

**FIGURE 2:**
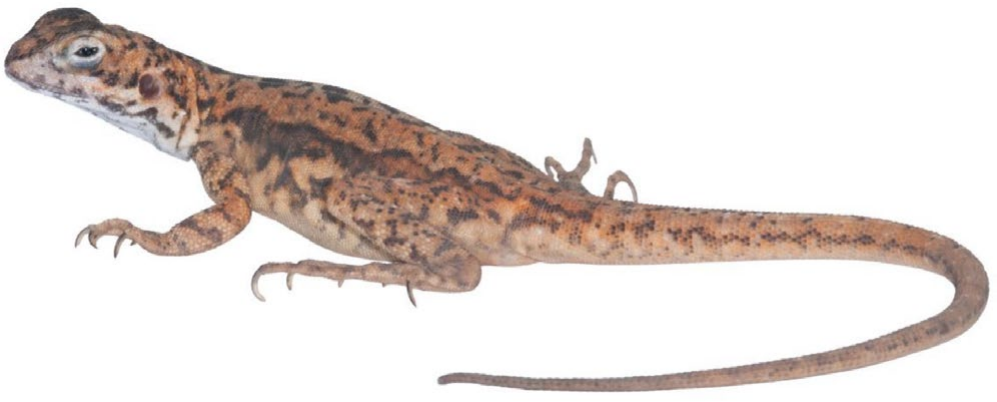
A female rusty dragon ( *Ctenophorus rufescens* ) that was collected from the wild as an adult in 2012 and lived in captivity for 12 years. We estimate this made her at least 13 years old at the time of her death. This is the longest known lifespan of any *Ctenophorus* dragon. This individual is now in the collection of the Australian Museum, Sydney, accession number AMS R.203166. Photo by Martin J. Whiting.

*Ctenophorus* dragons are unusual among reptiles as over half of the species with known lifespans are annuals. This split between annual and longer‐lived species appears not to be phylogenetically conserved but rather the product of multiple transitions between life history tactics as they relate to longevity. Furthermore, data from captivity indicate that species may be facultatively annual, and able to live longer than a year under ideal captive conditions, or obligately annual, living only 1 year no matter the conditions.

These are tantalising initial patterns, but the inferences we can draw are limited by the data currently available. We found longevity data from the wild for about 40% of *Ctenophorus* species and captive longevity data for about 14% of species, including the rusty dragon longevity record we report here. To better understand these unique life history shifts, we encourage anyone with records of the lifespans of *Ctenophorus* species to report them. Additional data on the *Ctenophorus* (and other) clades will enable us to better understand the degree to which longevity constrains life history in lizards.

## Author Contributions

**Daniel Hoops:** conceptualization (equal), data curation (lead), funding acquisition (lead), investigation (equal), methodology (equal), visualization (equal), writing – original draft (lead), writing – review and editing (equal). **Martin J. Whiting:** conceptualization (equal), investigation (equal), methodology (equal), resources (lead), visualization (equal), writing – review and editing (equal).

## Funding

This work was supported by grants to DH from The National Science and Engineering Council of Canada (CGSM‐393571‐2010, PGSD3‐415253‐2012) and The Government of Australia (APA#31/2011, IPRS#1182/2010).

## Conflicts of Interest

The authors declare no conflicts of interest.

## Data Availability

All data and code generated for this manuscript are available at the Open Science Framework repository for this manuscript, DOI: https://doi.org/10.17605/OSF.IO/8NU2X.
